# Secrecy Coding Analysis of Short-Packet Full-Duplex Transmissions with Joint Iterative Channel Estimation and Decoding Processes

**DOI:** 10.3390/s22145257

**Published:** 2022-07-14

**Authors:** Bao Quoc Vuong, Roland Gautier, Anthony Fiche, Mélanie Marazin, Cristina Despina-Stoian

**Affiliations:** 1 Univ Brest, CNRS, Lab-STICC, CS 93837, 6 Avenue Le Gorgeu, CEDEX 3, 29238 Brest, France; roland.gautier@univ-brest.fr (R.G.); anthony.fiche@univ-brest.fr (A.F.); melanie.marazin@univ-brest.fr (M.M.); cristina.despina@mta.ro (C.D.-S.); 2 School of Electrical Engineering, International University, Ho Chi Minh City 700000, Vietnam; 3 Vietnam National University, Ho Chi Minh City 700000, Vietnam; 4 Telecommunications and Information Technology Department, Military Technical Academy “Ferdinand I”, 050141 Bucharest, Romania

**Keywords:** security gap, channel coding scheme, physical layer security, self-jamming, feedback, blind channel estimation, semi-blind channel estimation

## Abstract

This paper studies the secrecy coding analysis achieved by the self-jamming technique in the presence of an eavesdropper by considering a short-packet Full-Duplex (FD) transmission developed based on iterative blind or semi-blind channel estimation and advanced decoding algorithms. Indeed, the legitimate receiver and eavesdropper can simultaneously receive the intended signal from the transmitter and broadcast a self-jamming or jamming signal to the others. Unlike other conventional techniques without feedback, the blind or semi-blind algorithm applied at the legitimate receiver can simultaneously estimate, firstly, the Self-Interference (SI) channel to cancel the SI component and, secondly, estimate the propagation channel, then decode the intended messages by using 5G Quasi-Cyclic Low-Density Parity Check (QC-LDPC) codes. Taking into account the passive eavesdropper case, the blind channel estimation with a feedback scheme is applied, where the temporary estimation of the intended channel and the decoded message are fed back to improve both the channel estimation and the decoding processes. Only the blind algorithm needs to be implemented in the case of a passive eavesdropper because it achieves sufficient performances and does not require adding pilot symbols as the semi-blind algorithm. In the case of an active eavesdropper, based on its robustness in the low region of the Signal-to-Noise Ratio (SNR), the semi-blind algorithm is considered by trading four pilot symbols and only requiring the feedback for channel estimation processes in order to overcome the increase in noise in the legitimate receiver. The results show that the blind or semi-blind algorithms outperform the conventional algorithm in terms of Mean Square Error (MSE), Bit Error Rate (BER) and security gap ($S_{g}$). In addition, it has been shown that the blind or semi-blind algorithms are less sensitive to high SI and self-jamming interference power levels imposed by secured FD transmission than the conventional algorithms without feedback.

## 1. Introduction

The development of future wireless technologies such as massive MIMO systems, machine type communications, millimeter wave transmissions, and especially the Internet of Things (IoT) has led to not only new challenges but also new opportunities in the 5G security domains [1]. In order to achieve enhanced security performances for the wireless communications, a better strategy is setting up based on the physical layer directly, which is a method that belongs to the information theory field. This category of security solutions, also known as Physical Layer Security (PLS), has recently emerged as a new security or additional security layer, especially in 5G networks and beyond [2,3]. The wiretap channel was first introduced by Wyner in 1975 and became fundamental in characterizing PLS problems, where the intended transmitter sends a message to the legitimate receiver while the passive eavesdropper tries to listen and decode this message [4]. A decade later, Ozarow and Wyner introduced the second type of wiretap channel, known as wiretap channel II, where the active eavesdropper not only listens to the intended transmitter but also transmits a jamming signal to the legitimate receiver [5]. Therefore, the presence of an active eavesdropper can more strongly destroy the reception behavior of the legitimate receiver than that of the passive eavesdropper in the secrecy of wireless communication links [6]. As a metric of PLS, the security gap was first introduced in [7], which is calculated as the ratio of the Bit Error Rate (BER) on the linear scale or the difference of the BERs on the log scale achieved by the legitimate receiver and the eavesdropper to ensure that the legitimate receiver can reliably receive the intended message and to maintain security throughout transmission.

Due to the improved spectral efficiency, Full-Duplex (FD) communication systems that simultaneously transmit and receive information using the same time-frequency channel resource became an essential approach in 5G and beyond communication networks, especially in IoT transmissions and green communications [8,9]. However, Self-Interference (SI) cancellation is still the biggest challenge of any FD system due to the channel estimation error caused by the complexity of the SI channel, particularly in the case of short-frame transmission [10,11,12]. The presence of SI will reduce the Signal-to-Noise Ratio (SNR) at the receiver and leads to low overall performance. Considering the PLS in FD transmission, the security approaches involving the simultaneous transmission of the self-jamming or Artificial Noise (AN) have attracted a huge research interest due to their robustness promising performance [13,14]. The self-jamming technique is usually used to make the interception and the correct message decoding impossible for the eavesdroppers, even if they have equivalent or better channel conditions than the legitimate receiver. Therefore, the self-jamming approach has been widely studied and extended in numerous schemes to enhance the PLS, i.e., the FD transceiver can simultaneously receive the intended message and broadcast the AN to degrade the eavesdropper channel [15,16,17]. The AN technique is also used for secure transmission in cognitive wiretap networks with FD receivers [18] or FD relay systems [19]. Usually, the PLS mechanism related to the self-jamming and AN has been studied assuming that the eavesdropper cannot estimate the wiretap channel or jamming channel based on the known training pilots and transmitted power [20]. However, a large number of training pilots are required to be involved, and it is still not a satisfying solution in terms of time, bandwidth, and power consumption, especially for short-frame FD transmission, because the training requires a huge number of data symbols to obtain a good second-order statistic of the received signal. Furthermore, channel secrecy capacity and transmission message reliability can be a problem for communications with finite block length or short-packet [6]. Therefore, the PLS on short-packet transmission has recently become an open area to be focused on in 5G and beyond, especially for IoT transmissions and green communications.

Furthermore, new radio channel coding schemes such as 5G Quasi-Cyclic Low-Density Parity Check (QC-LDPC) codes can also be chosen for the PLS problem due to their higher error correction performance and powerful decoding for both on and below the reliability threshold [1,7], as well as sufficient performance in ultra-reliable Low Latency Communication (uRLLC) in short-packet 5G transmission systems [21]. In recent years, many researchers have focused on secrecy channel coding techniques in the wiretap channel [22,23,24,25]. In particular, the authors in [23] evaluated the reliability and security over the flat and fast-fading Gaussian wiretap channel for the construction of various LDPC codes with the puncturing and scrambling techniques. Furthermore, the authors in [26] used the McEliece coding method based on LDPC Code to guarantee both information reliability between intended users and the security metric with respect to eavesdroppers in PLS. The authors in [24] also studied the combination of LDPC codes and AN by designing the scrambling matrix to reduce the probability of outage and improve PLS. Then, the authors in [25] proposed combining the LDPC codes at the transmitter and an iterative decoding algorithm at the receiver to reduce the security gap in the Gaussian wiretap channel. The results obtained show that their proposed scheme outperforms the punctured scheme in terms of the equivocation rate and the security gap. Last but not least, the authors in [27,28] proposed joint iterative blind and semi-blind algorithms for channel estimations and decoding processes in short-packet FD transmission. The results show that these algorithms outperform the conventional algorithms without feedback in terms of not only Mean Square Error (MSE) and BER performances but also processing time and computational complexity, which are suitable for IoT transmissions and green communications.

Therefore, in this paper, we propose and implement a new scheme that combines joint iterative channel estimation and decoding using 5G QC-LDPC codes with FD self-jamming of the legitimate receiver to enhance security and reliability, which means that the eavesdropper does not catch the information and the indented information is less affected or corrupted by the jamming signal, respectively, in two scenarios: a passive eavesdropper and an active eavesdropper. For the rest of this paper, the performance evaluations of the proposed algorithms are based on three metrics: MSE, BER, and security gap ($S_{g})$. The contributions of this paper can be summarized as follows:

- We evaluate a combination of self-jamming techniques with a joint iterative blind or semi-blind channel estimation and decoding for a FD short-packet transmissions in the cases of passive and active eavesdroppers, respectively;
- We characterize that the system developed based on the new proposed algorithms have better performance compared to the conventional algorithms without feedback in terms of security metrics;
- We point out that the legitimate receivers are less sensitive to self-interference as well as the jamming power from the eavesdropper in our approach.
- We emphasize that the proposed algorithms provides a higher robustness not only to the security and reliability factors but also to the power consumption by reducing the SNR at the legitimate receiver for decoding the message, which suits short-packet FD IoT transmissions and green communications.

The remainder of this paper is organized as follows. Section 2 briefly describes the general system model of the FD transceiver in the passive/active eavesdropper scenarios. The conventional schemes without feedback and security gaps are also mentioned in this section. Section 3 studies the application of the joint iterative blind channel estimation and decoding algorithm at the legitimate receiver in the case of passive eavesdroppers, with numerical results and comparisons with the conventional blind algorithm without feedback. Section 4 introduces the semi-blind algorithm for SI channel estimation and equalization processes in the legitimate receiver in case of active eavesdropper and simulation results. Finally, some highlights and conclusions will be discussed in Section 5. The notations in this paper are summarized in Table 1.

## 2. Full-Duplex Transceiver with Passive/Active Eavesdropper Transmission System

### 2.1. General System Model

**Figure 1:**
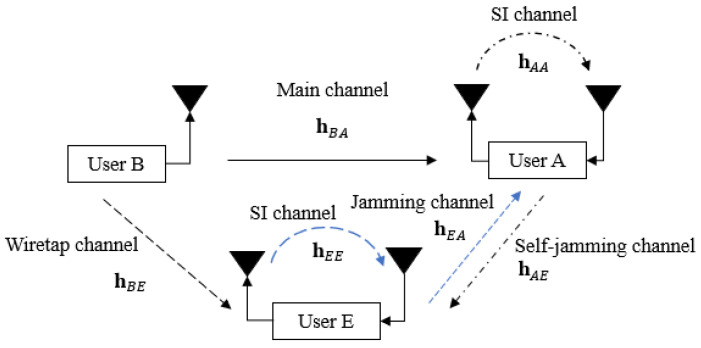
General system model.

We consider a short-packet FD transmission wiretap channel between three users, such as user B (transmitter), user A (legitimate receiver), and user E (eavesdropper), as shown in Figure 1, where the transmitter is equipped with only one antenna for transmission while the receiver and the eavesdropper are attached with one transmitter and one receiver antenna each to simultaneously receive the intended information message and transmit self-jamming or jamming signals. The 5G QC-LDPC codes, which are considered fundamental codes for short-packet uplink and downlink transmissions [29,30,31], are used in all transceivers.

At the transmitter, the (N,K) 5G QC-LDPC encoding process between the exponent parity check matrix H and the information bit sequence is based on the Gauss–Jordan elimination algorithm [32], where *K* and *N* denote the lengths of the information message and the code word message, respectively. Let us denote the channel gain between two users and the SI channel gain of itself as $h_{XY}$ and $h_{YY}$, respectively, in which X∈{A,B,E} and Y∈{A,E}, where A,B,E represent user A, user B, and user E, respectively. In this paper, the SI channel is modeled as quasi-static Rayleigh fading in the digital domain due to the assumption that the Line-of-Sight (LoS) component is fully suppressed by antenna and analog cancellation techniques, whereas the residual SI is the Non-Line-of-Sight (NLoS) component [8,33]. Note that $h_{XY}$ and $h_{YY}$ are i.i.d complex Gaussian random variables with CN(0,1) [34,35]. Moreover, the transmitted power of each user is denoted as $p_{X}$, where X∈{A,B,E}, and we further denote $w_{Y}$ as the complex background noise at user *Y* with $\mathrm{CN}(0,\sigma_{Y}^{2})$, where Y∈{A,E}. Based on the background noise as reference and without loss in generality, we further denote $\rho_{XY}=p_{X}/\sigma_{Y}^{2}$ and $\rho_{YY}=p_{Y}/\sigma_{Y}^{2}$ as the power-to-noise ratio provided by the self-jamming or jamming channel from user X to user Y and the SI channel at user Y, respectively. We also denote $SNR_{A}=p_{B}/\sigma_{A}^{2}$ and $SNR_{E}=p_{B}/\sigma_{E}^{2}$ as the SNR at user A and user E, where $\sigma_{A}^{2}$ and $\sigma_{E}^{2}$ are the noise powers at user A and user E, respectively.

### 2.2. Conventional Schemes without Feedback

**Figure 2:**
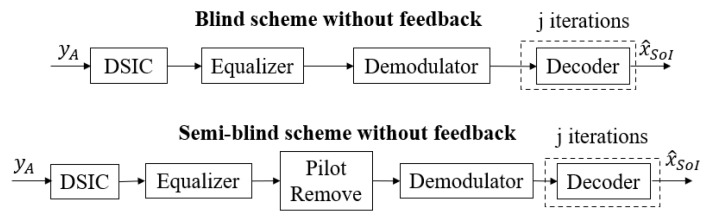
Conventional schemes without feedback.

The conventional blind scheme without feedback and the semi-blind scheme without feedback have been studied in [27,28], respectively, and are presented in Figure 2. At the receiver side, the received signal $y_{A}$ will pass through a Digital Self-Interference Cancellation (DSIC) process based on an adaptive filter with the Recursive Least Square (RLS) algorithm [36] to firstly estimate the SI channel and then reconstruct and cancel the SI component. Then, the residual signal will go to an equalizer to firstly estimate the intended channel and then obtain the equalized signal. In the semi-blind scheme without feedback, it is noted that the pilot symbols are attached to the information sequence at the transmitter side, and they are also used for the DSIC and equalizer processes. Then, they will be removed from the equalized signal, and this signal continuously goes to the demodulator to obtain the Log Likelihood Ratio (LLR) belief sequence. Finally, the Sum Product Algorithm (SPA) decoding algorithm [37,38] with an efficient message-passing schedule will be implemented in the 5G LDPC decoding process at user A and user E to reconstruct the binary input signal ${\hat{x}}_{SoI}$ of user B. The principle of SPA involves the message repetitively passing from the check nodes to the symbol nodes for guessing the transmitted bits from each other at each iteration *j* until it reaches the maximum number of iterations, $j_{max}$. For the rest of this paper, this DSIC process and decoding scheme are called blind scheme without feedback or semi-blind scheme without feedback, respectively.

In this paper, we assume the following hypotheses:

- In case of a passive eavesdropper, only blind channel estimation is used, where there is no knowledge about the channel state information at all communication users;
- In case of an active eavesdropper, both blind and semi-blind channel estimations, where all transceivers share a few pilot symbols, are mainly implemented;
- User E knows the parity check matrix H of user B and performs the SPA decoding mechanism; user E also uses an RLS algorithm in the DSIC process of user A in case of an active eavesdropper;
- Both user A and user E have equal computation capabilities, and the location of user E is close enough to user A to broadcast its jamming signal as well as to be attacked by the self-jamming signal from user A;
- The channel gains at the receiver and the eavesdropper are constant within a code word and change from one to another in fading channels;
- The impact of hardware impairments on the SI cancellation is not considered (which is outside the scope of this study but essential in practice). Moreover, the problem of the synchronization process between the transceivers is also not taken into account. Last but not least, the bit resolution of DAC/ADC is chosen to be high enough to bypass the effect of the quantization noise, i.e., larger than 6 bits for both DAC/ADC process. Alternatively, the oversampling should be applied in the ADC process if the green communication system and IoT applications are considered with low-bit ADC [39].

### 2.3. Security Gap

In the practical context of the wiretap channel when the short-packet is used for transmission, the typical BER performance criteria are usually used to ensure two aspects of performance such as reliability and secrecy conditions [23]. Let us denote $BER_{A}$ and $BER_{E}$ as the average BER of user A and user E, respectively. While $BER_{A,max}$ and $BER_{E,min}$ are the maximum BER that user A can achieve and the minimum BER that user E can obtain, respectively. The reliability condition holds when $BER_{A}\leq BER_{A,max}$, which means that the BER of user A should be maintained at a low value to enhance the reliability condition. Meanwhile, the security condition is achieved when $BER_{E}\geq BER_{E,min}$, which means that the BER of user E should remain at a sufficiently high value to guarantee the security.

According to [7,17], the security gap, which is the minimum difference of SNRs (in dB) required to guarantee the legitimate receiver security over the eavesdropper, is calculated as:

(1) $$S_{g}\left(\mathrm{dB}\right)=SNR_{A,min}-SNR_{E,max}$$

where $SNR_{A,min}$ is the minimum SNR corresponding to $BER_{A,max}$, where user A has to operate to make sure the BER is below some reliability thresholds, i.e., $BER_{A,max}=10^{-5}$, which is a sufficient level for practical applications [23]. Similarly, $SNR_{E,max}$ is the maximum SNR corresponding to $BER_{E,min}$ in which the BER of user E can approximately reach a threshold, that is, $BER_{E,min}=0.5$, which is called the security threshold because user E cannot exactly decode the information message in this region [7].

**Figure 3:**
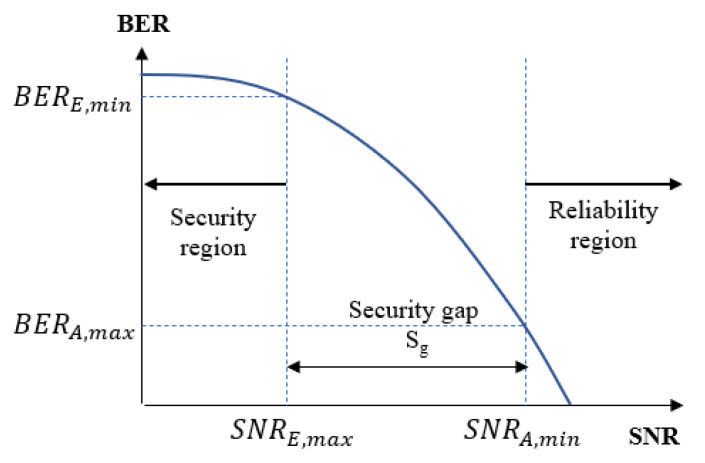
Security gap.

The graphical presentation of security gap is shown in Figure 3. In fact, the size of the security gap $S_{g}$ indicates the minimum cost of the difference in SNRs between user A and user E that maintains the possibility of secure communication, the higher values of $S_{g}$ will lead to a higher transmission cost. Therefore, the objective of this paper is to reduce the size of the security gap $S_{g}$ as much as possible. In particular, the SNR of user A, $SNR_{A}=p_{B}/\sigma_{A}^{2}$ (dB), on the main channel must be small enough to ensure that user A can correctly decode the information message from user B assuming the lowest possible power. In contrast, the SNR of user E, $SNR_{E}=p_{B}/\sigma_{E}^{2}$ (dB), on the wiretap channel must be as large as possible to guarantee that the self-jamming broadcasting from user A still affects the decoding process of user E.

Next, we will consider the first case with passive eavesdropper and the presence of a blind feedback algorithm.

## 3. Case I: Passive Eavesdropper

### 3.1. Passive Eavesdropper System Model

**Figure 4:**
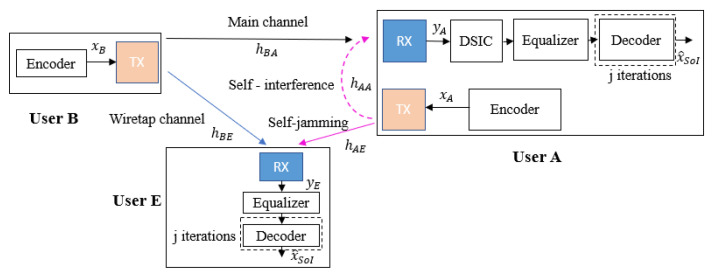
Blind scheme without feedback at user A in case of a passive eavesdropper.

**Figure 5:**
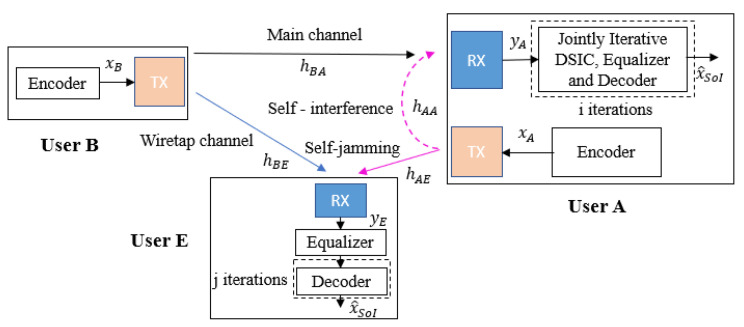
Blind feedback scheme at user A in case of a passive eavesdropper.

The wiretap channel system models with the use of FD self-jamming and a passive eavesdropper are shown in Figure 4 and Figure 5, where user A is operated in FD transmission mode to simultaneously receive the intended information message from user B and transmit the self-jamming signal to destroy the decoding ability of user E, while user E just tries to listen and decode the message from user B. The transmission strategy of the proposed scheme is as follows. User B wants to send his encoded message $x_{B}$ to the legitimate receiver user A through the main channel $h_{BA}$, while passive eavesdropper user E tries to listen and decode user B’s message through the wiretap channel $h_{BE}$. The received signals in the digital domain at user A and user E are given by the following:

(2) $$\begin{array}{cc} y_{A}\left[n\right] & =y_{BA}\left[n\right]+y_{AA}\left[n\right]+w_{A}\left[n\right]\\ & =\left(\sqrt{p_{B}}x_{B}*h_{BA}\right)\left[n\right]+\left(\sqrt{p_{A}}x_{A}*h_{AA}\right)\left[n\right]+w_{A}\left[n\right]; \end{array}$$

(3) $$\begin{array}{cc} y_{E}\left[n\right] & =y_{BE}\left[n\right]+y_{AE}\left[n\right]+w_{E}\left[n\right]\\ & =\left(\sqrt{p_{B}}x_{B}*h_{BE}\right)\left[n\right]+\left(\sqrt{p_{A}}x_{A}*h_{AE}\right)\left[n\right]+w_{E}\left[n\right]; \end{array}$$

where $w_{A}$ and $w_{E}$ are the complex Gaussian background noise of the receiver channel of user A and user E, with $\mathrm{CN}(0,\sigma_{A}^{2})$ and $\mathrm{CN}(0,\sigma_{E}^{2})$, respectively, and (∗) is the convolution operation.

The legitimate receiver user A obtains the signal $y_{A}$ and performs two possible decoding strategies to eliminate the SI component and obtain the estimation of the intended signal ${\hat{x}}_{SoI}$. First, it may use a classical blind scheme without feedback where the DSIC and decoding processes are independent, as presented in Figure 4. Second, it can use a more efficient scheme based on joint iterative blind channel estimation and decoding through feedback, as shown in Figure 5, which we call the blind feedback scheme. At the same time, user E also tries to listen to the transmission over the wiretap channel and only performs the equalization process and the classical SPA decoding process to obtain the original signal $x_{B}$.

Next, we will briefly describe the joint iterative blind channel estimation and decoding processes, which were studied in [27].

### 3.2. Blind Feedback Scheme

**Figure 6:**
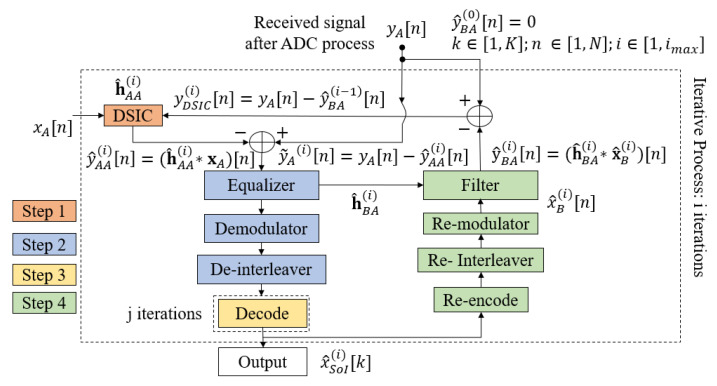
Joint iterative blind algorithm flowchart.

The conventional scheme without feedback with the RLS algorithm and the SPA decoding algorithm is an optimal estimation [40] and decoding algorithm, but with a high computational complexity [41], because it requires an updated LLR sequence and decoding for each iteration. It is not suitable for short-packet FD transmission due to the high estimation error of the SI channel [10] and power consumption in IoT applications and green communications due to the high latency of the 5G QC-LDPC decoder [42,43]. To overcome these drawbacks, the authors in [27] proposed a joint iterative algorithm for blind channel estimation and decoding, named the blind feedback scheme. The fundamental process of the blind feedback scheme is that the SI cancellation, intended channel estimation, and decoding processes of the desired signal can benefit from each other through the temporary decoding and feedback loop. Hence, the proposed scheme will only consider one iteration of SPA decoding ($j_{max}=1$) for each joint iteration *i*, called temporary decoding, and it will then perform the re-encoding, re-interleaving, and re-modulating to form a feedback loop of the intended signal in order to improve the SI cancellation process in the next joint iterations. This is continue until the system reaches the maximum number of joint iterations $i_{max}$. The proposed algorithm can not only decrease the processing time and computational complexity, but it can also improve the overall performance, which is illustrated in [27]. The flow chart of the proposed blind algorithm is described in Figure 6, which has four main steps and can be summarized as follows:

**Step 1:** The mixed signal $y_{A}$ at the receiver side is firstly used to estimate the SI channel ${\hat{h}}_{AA}$ and cancel the SI component based on the reference transmitted signal $x_{A}$;

**Step 2:** The residual signal ${\tilde{y}}_{A}$ received from Step 1 is continuously used to estimate the intended channel ${\hat{h}}_{BA}$ and obtain the equalized signal by an equalizer. Here, the *blind* channel estimation method is applied with no knowledge of the transmitting signal from the transmitter. Then, this equalized signal goes to the demodulator and de-interleaver to obtain the LLR belief information sequence.

**Step 3:** In this step, the estimation of the binary intended signal is achieved by using 5G QC-LDPC decoding with the SPA algorithm.

**Step 4:** When the maximum number of joint iterations ($i_{max}$) is not reached, the temporary message obtained from the previous Step is re-encoded, re-interleaved, and re-modulated. Then, this signal is filtered with the estimation version of the intended channel ${\hat{h}}_{BA}$ achieved in Step 2 to form the intended feedback signal ${\hat{y}}_{BA}$. Consequently, the intended feedback signal is used to temporarily remove the intended component from the received signal in order to optimize the SI channel estimation process for the next joint iteration.

### 3.3. Simulation Specifications

To evaluate the secrecy performance of our proposed schemes, MSE, BER, and security gap $S_{g}$ will be computed by using Monte Carlo simulations in MATLAB. For the rest of this paper, the MSE of the channel estimation in the intended receiver user A and the eavesdropper user E are given by [44]:

(4) $${\mathrm{MSE}}_{XX}=|h_{XX}-{\hat{h}}_{XX}{|}^{2},$$

and

(5) $${\mathrm{MSE}}_{XY}=|h_{XY}-{\hat{h}}_{XY}{|}^{2},$$

respectively.

For 5G QC-LDPC codes, the base graph matrix **BG2** [30] is implemented for all simulations. The SI channel and self-jamming or jamming channel are fixed with three taps based on Rayleigh distribution with CN(0,1). The intended main channel and wiretap are fixed with four taps and the power of each tap is according to the ITU–R channel model [45]. These channels are generated independently in each transmission frame. The simulation parameters of this paper are summarized in Table 2.

### 3.4. MSE Performances

#### 3.4.1. MSE at the Legitimate Receiver User A

**Figure 7:**
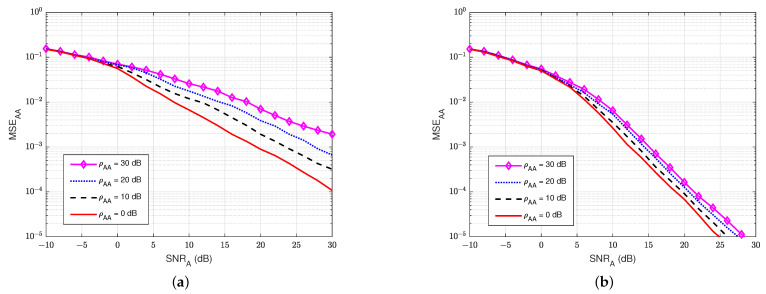
${MSE}_{AA}$ versus $SNR_{A}$ in case of passive eavesdropper: (**a**) Blind without feedback; (**b**) Blind feedback.

**Figure 8:**
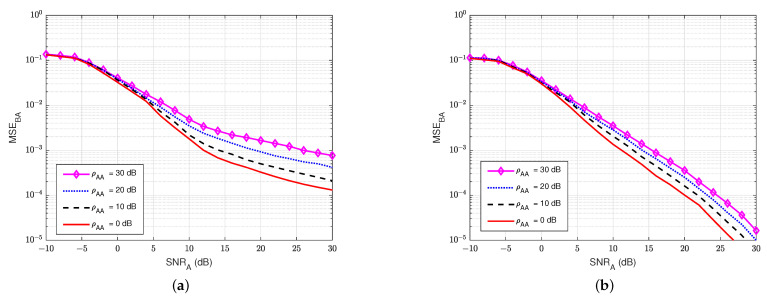
${MSE}_{BA}$ versus $SNR_{A}$ in case of passive eavesdropper: (**a**) Blind without feedback; (**b**) Blind feedback.

First, the MSEs of SI channel and main channel at user A are computed for different values of self-interference-to-noise ratio $\rho_{AA}$. For instance, Figure 7a,b show the MSEs of the SI channel versus $SNR_{A}$ of the legitimate receiver user A in the blind without feedback and blind feedback schemes, respectively. Similarly, Figure 8a,b illustrate the MSEs of the main channel versus the $SNR_{A}$ at user A in the blind without feedback and blind feedback schemes, respectively. It can be seen that MSEs increase significantly as the self-interference-to-noise ratio of user A ($\rho_{AA}$) increases, and the blind feedback scheme outperforms the scheme without feedback. It can also be observed that the increase in the self-interference-to-noise ratio of user A has less effect on the blind feedback scheme than the scheme without feedback. For example, maintaining ${\mathrm{MSE}}_{AA}$ at $10^{-3}$, when $\rho_{AA}$ increases from 0 to 30 dB, requires an increase of $SNR_{A}$ only around 2.5 to 3 dB in the blind feedback scheme. However, it requires an increase of roughly 10 dB in the scheme without feedback. Therefore, the use of the blind feedback scheme can improve the channel estimation processes at user A significantly.

#### 3.4.2. MSE at the Eavesdropper User E

**Figure 9:**
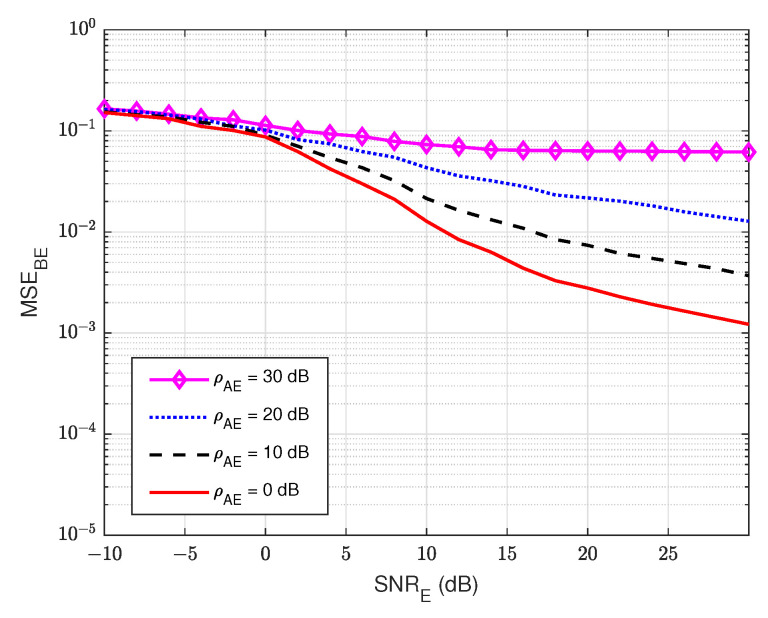
${MSE}_{BE}$ versus $SNR_{E}$ in case of passive eavesdropper.

Next, we also evaluate the MSE of the wiretap channel $h_{BE}$ versus the signal-to-noise ratio at the eavesdropper user E ($SNR_{E}$) for various values of the self-jamming-to-noise ratio from user A, $\rho_{AE}$. Based on Figure 9, it can be clearly observed that user E cannot estimate the wiretap channel well, especially in the case of a high value of the self-jamming-to-noise ratio of user A, i.e., when $\rho_{AE}$ increases higher than 10 dB. This behavior is due to the lack of knowledge of the reference signal of the transmitter as well as the power of the self-jamming signal from user A, which is much greater than the power of the intended signal. So, we can conclude that user E cannot accurately estimate the wiretap channel in passive mode.

### 3.5. BER Performances

#### 3.5.1. BER at the Legitimate Receiver User A

**Figure 10:**
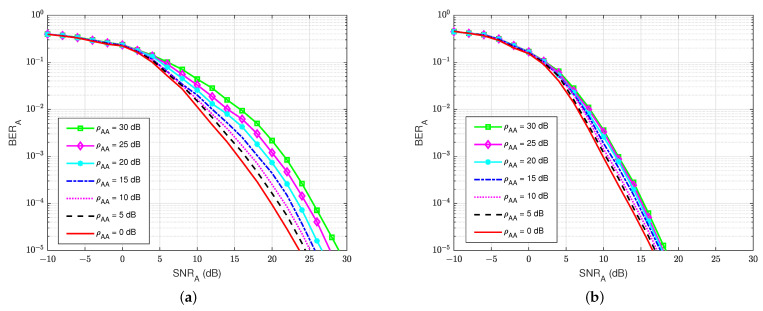
${BER}_{A}$ versus $SNR_{A}$ in case of a passive eavesdropper: (**a**) Blind without feedback; (**b**) Blind feedback.

The BER performances versus the $SNR_{A}$ of user A, for different values of the self-interference-to-noise ratio of user A ($\rho_{AA}$), are presented in Figure 10a,b for both the without feedback and blind feedback schemes, respectively. We can observe that the self-interference-to-noise ratio also significantly impacts the BER’s performance, i.e., the BER increases as the $\rho_{AA}$ increases, and the increase in the BER is bigger for larger $SNR_{A}$. It also shows an interesting result that when maintaining $BER_{A}=10^{-5}$ and increasing the self-interference-to-noise ratio $\rho_{AA}$ from 0 to 30 dB, the blind feedback scheme needs about 2 to 3 dB in $SNR_{A}$ to obtain that BER, while the scheme without feedback requires more than 5 dB in $SNR_{A}$ to achieve comparable results. Therefore, in the passive eavesdropper case, the increase in the self-interference-to-noise ratio has less effect on the blind feedback scheme in the BER performance at the legitimate receiver user A.

#### 3.5.2. BER at the Eavesdropper User E

**Figure 11:**
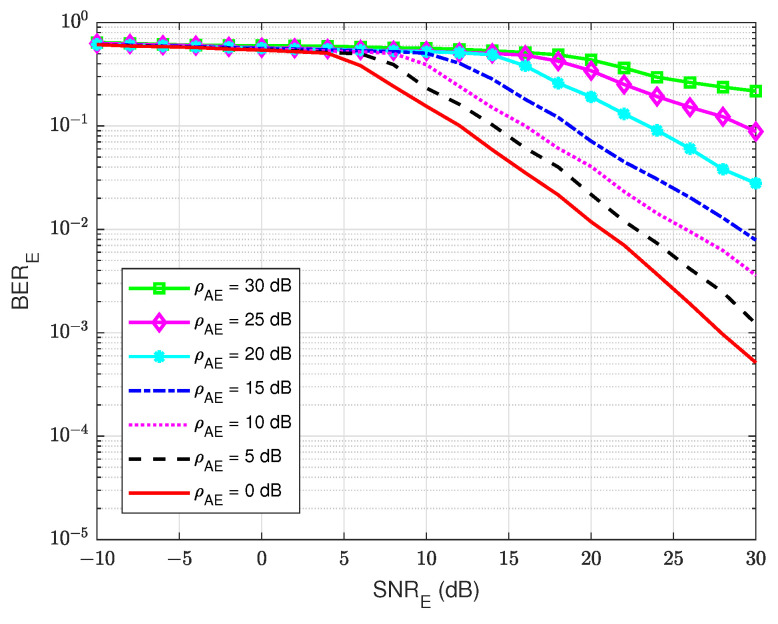
$BER_{E}$ versus the $SNR_{E}$ in case of a passive eavesdropper.

At the eavesdropper user E, the BER’s performances versus $SNR_{E}$ are also calculated to evaluate how much user E can decode the message sent from user B. For the rest of this paper, we have decided to keep the same BER ranges ($10^{0}$ to $10^{-5}$) without focusing on the useful ranges in order to allow for a visual comparison of the different schemes and especially the performance differences between legitimate user A and eavesdropper E. As shown in Figure 11, it is shown that the presence of a self-jamming signal from user A has a significant impact on the estimation and decoding processes of user E, regardless of the knowledge of the channel coding used for decoding. The best BER that user E can obtain is about $BER_{E}=10^{-3}$ at $SNR_{E}=30$ dB. Furthermore, when the self-jamming-to-noise ratio $\rho_{AE}$ is greater than 15 dB, user E almost cannot decode the intended message from user B. It can be explained that user A can estimate the SI channel well and cancel the SI component because user A has its generated self-jamming signal $x_{A}$ as reference. Moreover, applying the blind feedback scheme also improves the channel estimation and decoding processes, although user A also has no knowledge about the reference signal from user B. In contrast, user E has no knowledge about the reference signal of user B and the self-jamming signal of user A, and there is no interference cancellation mechanism applied; instead, it uses only the SPA decoding scheme to decode the intended message.

Therefore, user E cannot operate efficiently in the estimation and decoding processes. In summary, by applying the joint iterative estimation and decoding to the legitimate receiver, user A can significantly improve the secrecy reliability factor in FD wiretap transmission.

### 3.6. Security Gap Performance

On the one hand, there is an assumption concerning the relative positions of the various transmitters/receivers. In particular, for the case of user A and user E, it seems coherent and acceptable to consider that the powers of the background noises $\sigma_{A}^{2}$ and $\sigma_{E}^{2}$ are identical. On the other hand, under the assumption of channels without loss (unified mean deviations on all the paths) for $h_{AA}$ and $h_{AE}$, it is possible to simplify the notations and to denote in general the self-jamming power-to-noise ratio as $\rho_{SJ}$ for both self-interference $\left(\rho_{AA}\right)$ and self-jamming $\left(\rho_{AE}\right)$ channels. Since, in these conditions, we have $\rho_{SJ}=\rho_{AA}=\rho_{AE}$. The security gap $S_{g}$ is clearly related to the error rate achieved on the receiver side of user A and user E. In order to adapt to the practical applications, we set up $BER_{A,max}=10^{-5}$ and $BER_{E,min}=0.5$ for the maximum and minimum average errors that user A and user E can reach, respectively. Based on the results in Figure 10 and Figure 11, the minimum SNR at the legitimate user A, the $SNR_{A,min}$ and the maximum SNR at the eavesdropper user E, and $SNR_{E,max}$ to obtain $BER_{A,max}=10^{-5}$ and $BER_{E,min}=0.5$, respectively, can be pointed out. Then, these values are recorded corresponding to different levels of the general self-jamming power-to-noise ratio $\rho_{SJ}$. Finally, the security gap $S_{g}$ is calculated and summarized in Table 3.

**Figure 12:**
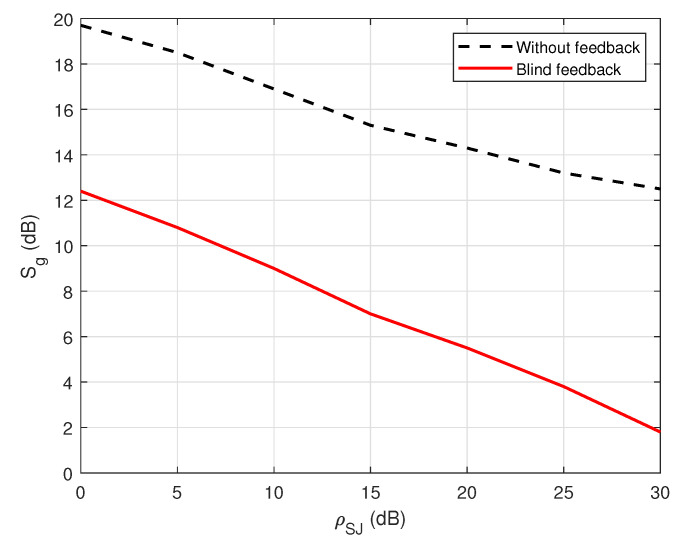
$S_{g}$ versus $\rho_{SJ}$ in the case of a passive eavesdropper.

Figure 12 shows the security gap versus the various values of the self-jamming power-to-noise ratio ($\rho_{SJ}$) in the case of blind without feedback and blind feedback at user A. The result shows that the increase in the self-jamming power-to-noise ratio $\rho_{SJ}$ leads to a decrease in the security gap $S_{g}$. For example, the security gap $S_{g}$ can be dramatically reduced from 7 to 10 dB when the blind feedback scheme is applied. Therefore, it obtains an important goal of the PLS, which is to maintain the security gap as small as possible. In summary, the use of joint iterative blind estimation and decoding at the legitimate receiver user A significantly reduces the security gap $S_{g}$ in FD wiretap transmission. Furthermore, when using the blind feedback scheme, the $SNR_{A}$ of user A is reduced when performing channel estimation or decoding messages, compared with the blind scheme without feedback, which emphasizes that the system not only maintains security but also enhances power consumption by reducing the transmission power.

In the next section, we will consider the second case, where user E can also send their jamming message to destroy the reception and decoding processes of user A, which we refer to as an active eavesdropper.

## 4. Case II: Active Eavesdropper

### 4.1. Active Eavesdropper System Model

**Figure 13:**
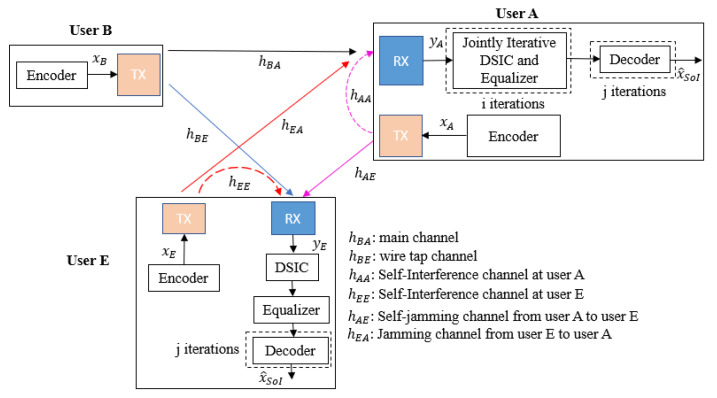
Wiretap Full-Duplex transmission with self-jamming in case of an active eavesdropper.

The wiretap channel system model with the use of FD self-jamming and an active eavesdropper is shown in Figure 13.

In this case, both user A and user E operate in the FD transmission mode to simultaneously receive the intended information message from user B and transmit the self-jamming or jamming signal to other users. In particular, user B wants to send his encoded message $x_{B}$ to the legitimate receiver user A by the main channel, while the eavesdropper user E not only tries to listen to and decode user B’s message by the wiretap channel but also simultaneously broadcasts their jamming signal to user A. Consequently, the received signals in the digital domain at user A and user E are given by:

(6) $$\begin{array}{cc} y_{A}\left[n\right] & =y_{BA}\left[n\right]+y_{AA}\left[n\right]+y_{EA}\left[n\right]+w_{A}\left[n\right]\\ & =\left(\sqrt{p_{B}}x_{B}*h_{BA}\right)\left[n\right]+\left(\sqrt{p_{A}}x_{A}*h_{AA}\right)\left[n\right]+\left(\sqrt{p_{E}}x_{E}*h_{EA}\right)\left[n\right]+w_{A}\left[n\right]; \end{array}$$

and

(7) $$\begin{array}{cc} y_{E}\left[n\right] & =y_{BE}\left[n\right]+y_{EE}\left[n\right]+y_{AE}\left[n\right]+w_{E}\left[n\right]\\ & =\left(\sqrt{p_{B}}x_{B}*h_{BE}\right)\left[n\right]+\left(\sqrt{p_{E}}x_{E}*h_{EE}\right)\left[n\right]+\left(\sqrt{p_{A}}x_{A}*h_{AE}\right)\left[n\right]+w_{E}\left[n\right], \end{array}$$

respectively.

It can be seen that the signal-to-noise ratio at user A is reduced due to the impact of the jamming signal from user E, which leads to an increase in noise at the receiver of user A. Therefore, in addition to the proposed blind feedback scheme, the joint iterative SI channel estimation and equalization processes with the semi-blind algorithm, which have been studied in [28], should be used at user A in order to eliminate the SI component and estimate the intended signal ${\hat{x}}_{SoI}$ because the proposed semi-blind algorithm shows its robustness in the low region of the SNR, compared to the blind algorithm. Indeed, the principle of this algorithm is to use at least four pilot symbols between the transceivers (which is a sufficient number of pilot symbols, as shown in [28]) to perform the channel estimation processes as well as the feedback loop. At the receiver side of user E, in order to distinguish the decoding behavior of the legitimate receiver (user A) and the eavesdropper (user E) and to also show the robustness of two proposed feedback schemes over the conventional schemes without feedback, user E will only use the blind scheme without feedback and the semi-blind scheme without feedback. In case of the semi-blind scheme without feedback, it is also assumed that four pilot symbols are observed by user E.

Next, we will briefly mention and summarize the joint iterative semi-blind channel estimation and equalization processes and name it the semi-blind feedback scheme.

### 4.2. Semi-Blind Feedback Scheme

In the case of a passive eavesdropper, it is shown that the performance is better when using the blind feedback scheme. However, in the case of an active eavesdropper, the presence of a jamming signal from user E leads to significant destruction of the reception behavior of user A. Therefore, small sharing of known symbols or pilot symbols between user B and user A should be established to guarantee the reliability and security of transmissions.

**Figure 14:**
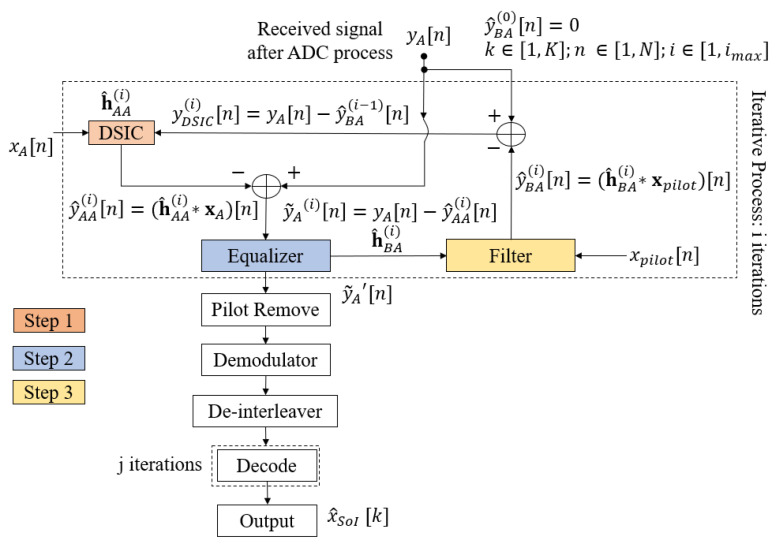
Joint iterative semi-blind algorithm flow chart.

The processing flowchart of the semi-blind algorithm is presented in Figure 14. In general, it is nearly similar to the joint iterative blind feedback algorithm in Section 3.2, except that the temporary decoding and encoding processes are skipped. Instead, the known pilot symbols, which are added to the information sequence on the transmitter side, are used to form the intended signal and the feedback loop. In particular, for i=1 (first iteration of the iterative algorithm), a first SI cancellation and intended channel estimation are performed for all symbols in order to overcome a larger number of errors and achieve a sufficient level of convergence. When $i\in [2,i_{max}]$, the known pilot symbols $x_{pilot}=4$ (symbols) are used to form the feedback loop. When it reaches the maximum number of iterations ($i=i_{max}$), the algorithm is stopped, the SI component can be canceled, and the equalized signal can be fully achieved by the estimation versions of the SI channel and the intended channel. After that, the known pilot symbols are suppressed, and the equalized signal will undergo the demodulation, de-interleaver, and decoding processes to obtain the final decoded message. Here, it is noticed that the SPA decoding algorithm also performs only one iteration ($j_{max}=1)$ in the decoding step when the system achieves the best channel estimation ($i=i_{max}$).

The proposed semi-blind algorithm can be summarized in three steps:

**Step 1:** The received signal $y_{A}$ is used to estimate the SI channel ${\hat{h}}_{AA}$ and to cancel the SI component based on the reference transmitted signal $x_{A}$;

**Step 2:** The residual signal ${\tilde{y}}_{A}$ after step 1 will go to an equalizer to estimate the intended channel ${\hat{h}}_{BA}$;

**Step 3:** Using pilot symbols $x_{pilot}$ that are added to the information sequence on the transmitting side, a feedback loop is created with the estimation version of the intended channel ${\hat{h}}_{BA}$ to form ${\hat{y}}_{BA}$. This signal is passed to the subtraction process from the received signal and performs the next joint iterations.

Next, we will introduce the performance in terms of the MSE, BER, and security gap $S_{g}$ in the case of an active eavesdropper.

### 4.3. Mean Square Error (MSE) Performance

#### 4.3.1. MSE at the Legitimate Receiver User A

**Figure 15:**
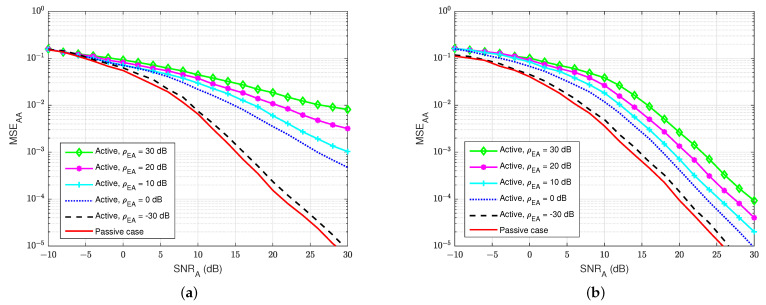
${MSE}_{AA}$ versus $SNR_{A}$, $\rho_{AA}=30$ dB in case of active eavesdropper: (**a**) Blind feedback; (**b**) Semi-blind feedback.

**Figure 16:**
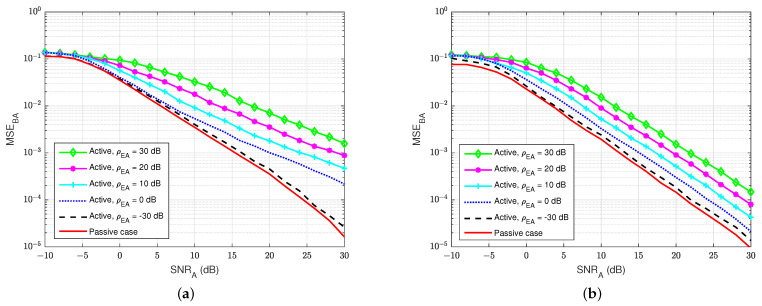
${MSE}_{BA}$ versus $SNR_{A}$, $\rho_{AA}=30$ dB in case of an active eavesdropper: (**a**) Blind feedback; (**b**) Semi-blind feedback.

First of all, Figure 15 and Figure 16 illustrate the MSEs of the SI channel and the main channel at user A for the blind feedback scheme and the semi-blind feedback scheme, respectively, versus $SNR_{A}$ for different power values of the jamming-to-noise ratio $\rho_{EA}$ broadcast from user E, while the self-interference-to-noise ratio at user A, $\rho_{AA}$, is fixed at 30 dB. It can be seen that the presence of the jamming signal from user E significantly impacts the SI channel estimation at user A, where it increases the noise level at the receiver side at user A, compared with the passive case. Indeed, the gain between each MSE’s curve is bigger than in the passive case, whatever the algorithm used, which means that the system requires higher $SNR_{A}$ to estimate the channel. Furthermore, the semi-blind feedback scheme outperforms the blind feedback scheme, i.e, it converges faster to the error floor and achieves better results than the blind feedback scheme because the traces of four pilot symbols is used. Therefore, using the semi-blind algorithm can improve the channel estimation processes and reduce the impact of the jamming signal from the eavesdropper.

#### 4.3.2. MSE at the Eavesdropper User E

**Figure 17:**
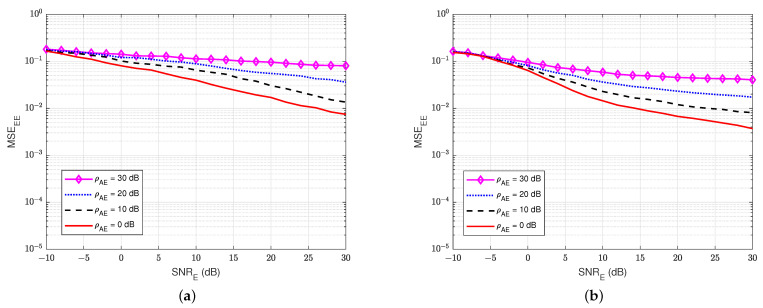
${MSE}_{EE}$ versus $SNR_{E}$, $\rho_{EE}$ = 30 dB in case of an active eavesdropper. (**a**) Blind without feedback; (**b**) Semi-blind without feedback.

**Figure 18:**
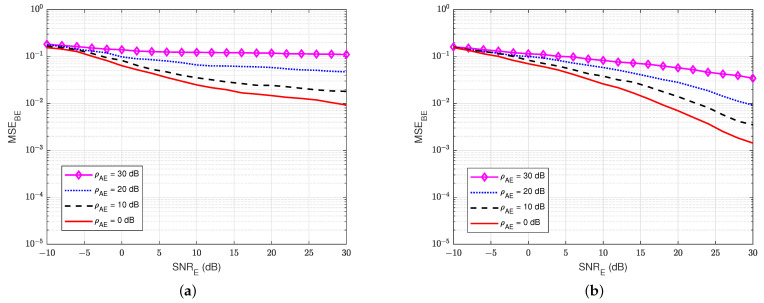
${MSE}_{BE}$ versus $SNR_{E}$, $\rho_{EE}$ = 30 dB in case of an active eavesdropper: (**a**) Blind without feedback; (**b**) Semi-blind without feedback.

Next, Figure 17 and Figure 18 show the MSEs of the SI channel $h_{EE}$ and the wiretap channel $h_{BE}$ versus $SNR_{E}$ at the eavesdropper user E for various values of the self-jamming-to-noise ratio from user A, $\rho_{AE}$. The self-interference-to-noise ratio at user E, $\rho_{EE}$, is fixed at 30 dB. It can be clearly observed that user E cannot estimate the wiretap channel and the SI channel well, especially if there is a high self-jamming-to-noise ratio from user A, i.e., $\rho_{AE}$ increases higher than 20 dB. So, the self-jamming signal provided by user A significantly influences the receiver side of user E, where user E cannot perform the wiretap channel estimation well in active mode, although user E also knows the pilot symbols. Moreover, the power of the combination of the self-jamming of user A and the SI component at user E is also higher than the power level of the intended message from user B. Therefore, the blind scheme without feedback and the semi-blind scheme without feedback, which are applied to user E, cannot estimate the channels well.

### 4.4. Bit-Error-Rate (BER) Performance

#### 4.4.1. BER at the Legitimate Receiver User A

**Figure 19:**
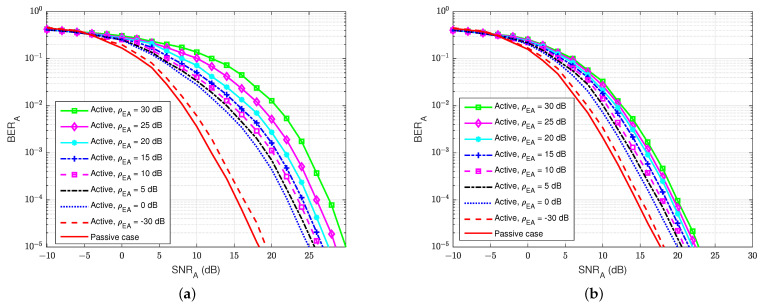
$BER_{A}$ versus $SNR_{A}$, $\rho_{AA}=30$ dB in case of an active eavesdropper: (**a**) Blind feedback; (**b**) Semi-blind feedback.

The BER performances versus $SNR_{A}$ at user A for different values of the jamming-to-noise ratio from user E ($\rho_{EA}$) are illustrated in Figure 19a,b for both the blind feedback scheme and the semi-blind scheme at user A, respectively. The self-interference-to-noise ratio at user A ($\rho_{AA}$) is set at 30 dB. We can observe that the BER increases as the jamming-to-noise ratio of user E ($\rho_{EA}$) increases, and the increase in the BER is bigger for larger $SNR_{A}$ values compared with the passive case. We can also remark that the semi-blind scheme is less sensitive to the jamming from user E than the blind feedback scheme, and it also converges faster to the error floor than the other. In particular, when maintaining $BER_{A}=10^{-5}$ and increasing the jamming-to-noise ratio $\rho_{EA}$ from 0 to 30 dB, the blind feedback scheme needs about 5 dB in $SNR_{A}$, while the semi-blind feedback scheme requires only 2.5 to 3 dB to reach that result. Therefore, the semi-blind feedback scheme is suitable in the case of an active eavesdropper because the increase in jamming power from the active user E has less influence on the BER performance at the legitimate receiver user A. In fact, it can considerably improve the reliability factor of secrecy in FD wiretap transmission in the case of an active eavesdropper.

#### 4.4.2. BER at the Eavesdropper User E

**Figure 20:**
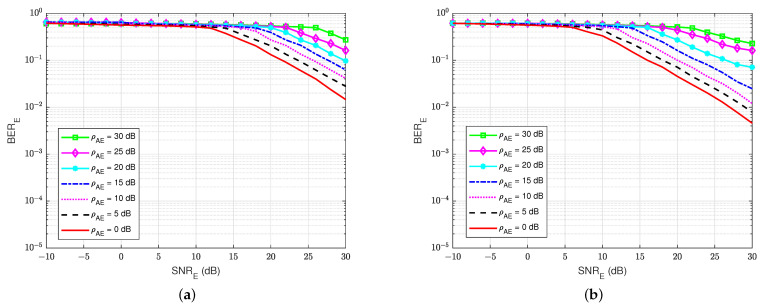
$BER_{E}$ versus $SNR_{E}$, $\rho_{EE}=30$ dB in case of an active eavesdropper: (**a**) Blind without feedback; (**b**) Semi-blind without feedback.

At the active eavesdropper user E, the BER performances versus $SNR_{E}$ are also calculated to evaluate the amount of the message that user E can decode. As shown in Figure 20, it can be seen that the combination of both the jamming signal from user A and the self-interference component at user E themself has a major impact on the estimating and decoding process of user E. This is because the combined power of these two signals is larger than the power of the intended signal, and user E only uses the blind or semi-blind scheme without feedback for channel estimation and decoding, regardless of the knowledge of channel coding used for decoding and the four pilot symbols. The best BER that user E can obtain is about $BER_{E}=10^{-2}$ at $SNR_{E}=30$ dB, corresponding to the lowest level of the self-jamming-to-noise ratio from user A, $\rho_{AE}=0$ dB. Consequently, when the power of the self-jamming signal from user A increases, user E needs a very large $SNR_{E}$ to decode the intended message from user B.

**Figure 21:**
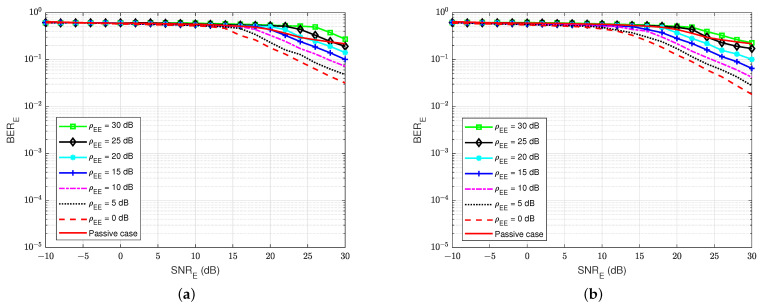
$BER_{E}$ versus $SNR_{E}$, $\rho_{AE}=30$ dB in case of an active eavesdropper: (**a**) Blind without feedback; (**b**) Semi-blind without feedback.

Furthermore, Figure 21 shows the BER of user E versus the $SNR_{E}$ for various values of the self-interference-to-noise ratio of itself, $\rho_{EE}$, while the self-jamming-to-noise ratio of user A, $\rho_{AE}$ is fixed at 30 dB. It shows that if user E tries to increase the power of the jamming signal that is sent to user A, it leads to an increase in their BER because of the increase in the self-interference-to-noise ratio $\rho_{EE}$. Although SI can be suppressed by the knowledge of the SI signal by the classical DSIC process, the interference from the self-jamming signal from user A still significantly impacts the blind scheme without feedback and the semi-blind scheme without feedback. It looks like the case of a passive eavesdropper when user E cannot suppress the interference from the jamming signal of user A well. However, the BER of user A is less sensitive to the increased power of user E, especially for the semi-blind feedback scheme, as shown in Figure 19.

Therefore, it can be concluded that user E cannot decode the message well, regardless of using the blind scheme without feedback or the semi-blind scheme without feedback.

### 4.5. Security Gap Performance

Considering the same assumptions that have been made for background noises and propagation channels in the case of a passive eavesdropper in Section 3.6, it is also possible to simplify the notations and to denote in general the self-jamming power-to-noise ratio as $\rho_{SJ}$ for both self-jamming $\left(\rho_{AE}\right)$ and jamming $\left(\rho_{EA}\right)$ channels. Since, in these conditions, we have $\rho_{SJ}=\rho_{AE}=\rho_{EA}$, adapting for practical applications, we also set $BER_{A,max}=10^{-5}$ and $BER_{E,min}=0.5$ for the maximum and minimum average errors that user A and user E can obtain, respectively. According to the results in Figure 19 and Figure 20, in order to achieve $BER_{A,max}=10^{-5}$ and $BER_{E,min}=0.5$, the minimum SNR at the legitimate user A, $SNR_{A,min}$, and the maximum SNR at the eavesdropper user E, $SNR_{E,max}$, can be pointed out for different values of the general self-jamming power-to-noise ratio ($\rho_{SJ}$) and for different decoding schemes at user A and user E. Then, the security gap $S_{g}$ is calculated based on $SNR_{A,min}$ and $SNR_{E,max}$ and summarized in Table 4 and Table 5 when using the blind scheme without feedback and the semi-blind scheme without feedback at user E, respectively.

**Figure 22:**
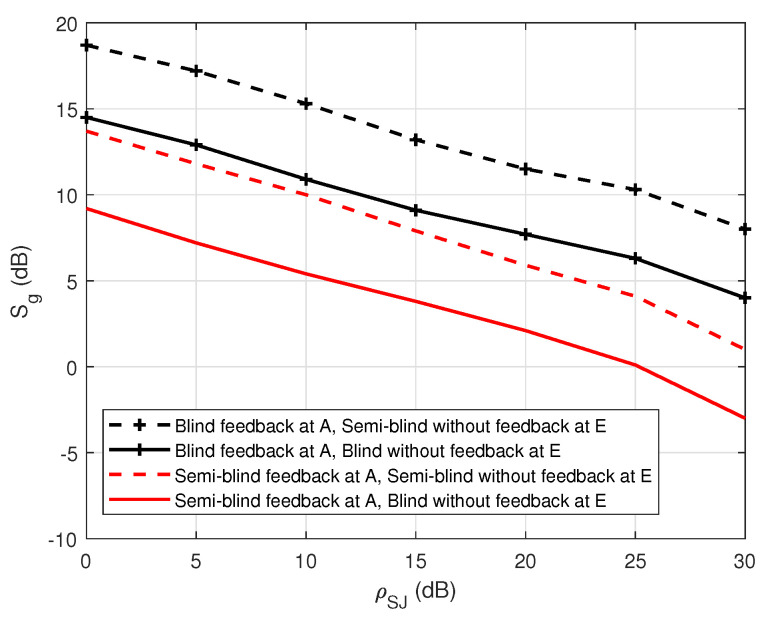
$S_{g}$ versus $\rho_{SJ}$ in case of an active eavesdropper.

Figure 22 shows the comparison of the security gap $S_{g}$ with the various values of the general self-jamming power-to-noise ratio, $\rho_{SJ}$, between the application of the blind feedback scheme and the semi-blind feedback scheme on the decoding side of user A. It indicates that the increase in the self-jamming power-to-noise ratio $\rho_{SJ}$ leads to a decrease in the security gap $S_{g}$ for all cases. The proposed semi-blind feedback scheme also allows for reducing the security gap $S_{g}$ from about 5 to 7 dB compared to the blind feedback scheme, regardless of the use of the blind or semi-blind scheme without feedback in user E. Therefore, it can keep the security gap as small as possible, which is the most important factor in PLS. Furthermore, the $SNR_{A}$ of user A is reduced when performing channel estimation or decoding the message using the semi-blind feedback scheme, compared to the blind feedback scheme, which means that the system not only guarantees the security factor but also improves power consumption.

## 5. Conclusions

The secrecy analysis of FD short-packet transmission in a wiretap channel for both passive and active eavesdroppers has been implemented, subject to the constraints of the MSE, BER, security gap $S_{g}$. This paper highlights that the presence of a jamming signal has a major effect on the reliability and security factors in PLS. To deal with this effect, a joint iterative SI channel estimation, propagation channel estimation, and decoding algorithm in FD transmissions via feedback were applied at the legitimate receiver, including blind feedback or semi-blind feedback schemes in the case of passive and active eavesdroppers, respectively. The numerical results presented show that the proposed algorithms, such as the blind feedback scheme in the passive case and the semi-blind feedback scheme in the active case, outperform the conventional algorithms without feedback, where the security gap $S_{g}$ is significantly reduced. Moreover, it can be noticed that the blind feedback scheme in the case of a passive eavesdropper and the semi-blind feedback scheme in the case of an active eavesdropper are less sensitive to the increase in self-jamming power. Moreover, the SNR of the legitimate receiver is reduced when applying the proposed schemes to decode the intended message, which means that the system not only ensures the security factor well, but it also significantly improves the power consumption by reducing the transmitting power. It is also noted that the proposed blind and semi-blind algorithms have better performances in terms of processing time and computational complexity, which are shown in [27,28]. Therefore, the presence of joint iterative estimation and decoding with blind and semi-blind algorithms at the legitimate receiver is highly recommenced to enhance the security of FD wiretap transmission, especially in short-packet transmission-specific to IoT applications and green communications.

## 6. Future Works

In the near future, several interesting investigations should be established in the context of the physical layer security field, especially in FD short-packet transmission. First, the location of the eavesdropper will be considered to emphasize the outperformance of the proposed algorithm with the conventional algorithm. Moreover, a hardware implementation based on Software-Defined Radio (SDR) will be considered to emphasize the performance of the proposed schemes in realistic transmission scenarios for IoT applications and green communications.

## Figures and Tables

**Table 1:** List of Notations.

Notations	Meaning
*K*, *N*, *R*	Information length, code word length, and code rate
$x_{X}$, $y_{X}$	Transmitted signal vector and received signal vector at user X
$h_{XY}$	Channel gain vector between X and Y
$h_{XX}$	Self-interference channel gain vector at user X
$y_{XX}$	Self-interference signal vector at user X
$y_{XY}$	Receiving signal vector that transmitted from user X to user Y
$\hat{x}$	Estimated signal vector
$\tilde{y}$	Residual signal vector
$x_{pilot}$	Pilot symbols vector
$SNR_{Y}$	Signal-to-noise ratio at user Y
$p_{Y}$	Transmitting power of user Y
$\sigma_{Y}^{2}$	Noise power at user Y
$w_{Y}$	Background noise at user Y
$\rho_{XY}$	Self-jamming power-to-noise ratio from user X to user Y
$\rho_{YY}$	SI power-to-noise ratio at user Y
λ	Forget factor of the RLS algorithm
*i*	Index of joint iterative iterations
*j*	Index of 5G QC-LDPC decoding iterations
*k*	Index of signal in the binary domain
*n*	Index of signal in the discrete time domain

**Table 2:** Simulation Specifications.

Parameter	Value
Number of transmission frames	$10^{6}$
Number of information bits and code word bits (K,N)	(128, 256)
Code rate *R*	1/2
Modulation scheme	QPSK
SI channel taps $h_{AA},h_{EE}$	3
Self-jamming channel taps $h_{AE}$	3
Jamming channel taps $h_{EA}$	3
Main channel taps $h_{BA}$	4
Wiretap channel taps $h_{BE}$	4
Number of pilot symbols in semi-blind scheme	4
Index of iterations $\left(i_{max},j_{max}\right)$ for scheme with feedback	(4,1)
Index of iteration $j_{max}$ for scheme without feedback	20

**Table 3:** Security gap $S_{g}$ in case of passive eavesdropper.

$\rho_{\mathrm{SJ}}$	${\mathrm{SNR}}_{E,\max}$	Without Feedback at User A		Blind Feedback at User A	
		${\mathrm{SNR}}_{A,\min}$	$S_{g}$	${\mathrm{SNR}}_{A,\min}$	$S_{g}$
0	4.1	23.8	19.7	16.5	12.4
5	5.8	24.5	18.7	16.8	10.8
10	8.1	25.1	17	17.2	9.1
15	10.3	25.6	15.3	17.6	7.3
20	12.4	26.7	14.3	17.9	5.5
25	14.3	27.5	13.2	18.1	3.8
30	16.6	29	12.4	18.3	1.7

**Table 4:** The security gap when applying the blind scheme without feedback at user E.

$\rho_{\mathrm{SJ}}$	Blind without Feedback at User E	Blind Feedback at User A		Semi-Blind Feedback at User A	
	${\mathrm{SNR}}_{E,\max}$	${\mathrm{SNR}}_{A,\min}$	$S_{g}$	${\mathrm{SNR}}_{A,\min}$	$S_{g}$
0	10.7	24.9	14.2	19.9	9.2
5	12.8	25.7	12.9	20.3	7.5
10	15.4	26.3	10.9	21	5.6
15	17.5	26.8	9.3	21.5	4
20	19.9	27.6	7.7	22	2.1
25	22.2	28.5	6.3	22.3	0.1
30	25.6	29.8	4.2	22.8	−2.8

**Table 5:** The security gap when applying the semi-blind scheme without feedback at user E.

$\rho_{\mathrm{SJ}}$	Semi-Blind without Feedback at User E	Blind Feedback at User A		Semi-Blind Feedback at User A	
	${\mathrm{SNR}}_{E,\max}$	${\mathrm{SNR}}_{A,\min}$	$S_{g}$	${\mathrm{SNR}}_{A,\min}$	$S_{g}$
0	6.2	24.9	18.7	19.9	13.7
5	8.2	25.7	17.5	20.3	12.1
10	10.8	26.3	15.5	21	10.2
15	13.4	26.8	13.4	21.5	8.1
20	16.1	27.6	11.5	22	5.9
25	18.2	28.5	10.3	22.3	4.1
30	21.5	29.8	8.3	22.8	1.3

## Data Availability

The data presented in this study are available on request from the corresponding author.

## Abbreviations

The following abbreviations are used in this manuscript:

3GPP	The Third-Generation Partnership Project
5G	The Fifth Generation
ADC	Analog-to-Digital Converter
BER	Bit Error Rate
DAC	Digital-to-Analog Converter
DSIC	Digital Self-interference Cancellation
FD	Full-Duplex
IoT	Internet of Things
ITU	International Telecommunication Union
LLR	Log Likelihood Ratio
LoS	Line-of-Sight
MSE	Mean Square Error
NLoS	Non-Line-of-Sight
PLS	Physical Layer Security
QC-LDPC	Quasi-Cyclic Low-Density Parity Check
QPSK	Quadature Phase Shift Keying
RF	Radio Frequency
RLS	Recursive Least Square
SDR	Software-Defined Radio
SI	Self-Interference
SPA	Sum Product Algorithm
uRLLC	Ultra-reliable Low-Latency Communication
